# Doctors' Fees and Nursing Homes

**Published:** 1912-09-07

**Authors:** Fair Game


### The Editor's Letter-Box.

### Doctors' Fees and Nursing Homes.

*To the Editor of* The Hospital.

Sir,—Having been abroad I have only just seen your issue of August 17 containing an article entitled " Doctors' Fees and Nursing Homes," in which you criticise a letter of mine that appeared in the *Times* of August 12.

You state that it is difficult to judge whether there is substantial foundation for my allegation that the doctor paid me unnecessary visits, as I do not give any particulars of my illness; and you suggest that possibly I stood in graver danger than I knew of, and may have received full value for my forty-two guineas. When I tell you that I was suffering from nothing more serious than the discomfort caused by slight varicose veins in the legs, you will recognise that my condition was not such as to require unremitting attention. And, further, when I explain that the treatment, which never varied throughout six weeks, consisted simply of hard massage twice a day and a plain diet, you will appreciate that the doctor's numerous visits can hardly have been due to the difficulties of my case.

But, even assuming that this close observation was desirable in my interest, is it conceivable that all his patients in the home should require the doctor's attention precisely as often and on exactly the same days as myself? The fact that Tuesdays, Thursdays, and Saturdays were known in the home as "Dr. X.'s days" points to one of two conclusions—either that the necessities of his patients demanded the doctor's presence week after week on those particular days and no others, or that his visits were a matter of unvarying routine. The latter conclusion seems to me to be irresistible.

You take exception in your article to my denunciation of "the system which enables a doctor to keep you for a prolonged period in a nursing home of his own choosing." You say there is no such system. If you will allow me space in your columns I think I can satisfy you that such a system does exist, and that it affords those who practice it facilities for making money which are denied to their more scrupulous colleagues. It is set in motion somewhat as follows: You are recommended to a certain doctor, who tells you that you will derive much benefit if you will submit to treatment in a nursing home for about six weeks: you suggest that on grounds of comfort and economy you would prefer to undergo the treatment in your own house; the doctor raises objections of a technical character, and shows a strong prejudice in favour of the nursing home; so, as you are anxious to obtain the full value of the treatment, you decide to enter the home he recommends. The "system" has now begun to work. It all goes like clockwork in the home. Everyone there has long experience of the stereotyped treatment which the doctor finds so beneficial to his patients. The cook is an expert in her duty; no one could make Dr. Schmidt's Diet less repulsive or Diet No. 1 less unappetising. The masseur flits from room to room pursuing his weary task. The nurses know exactly what to do as the treatment follows its accustomed course. The doctor comes three mornings a week with unfailing regularity and visits all his patients. He converses pleasantly on the topics of the hour, or on matters which concern himself but are of singularly little interest to a patient who wants to talk about his own health. And when business begins and you hope your symptoms are going to be discussed and given that careful consideration which they merit, you are told you are going on very well; that you must occupy your mind with subjects other than your health; that the same treatment will continue—and the door closes.

So much for the daily working of the system.

Now, Sir, let me deal with your contention that there can be no "system" because "no doctor can 'keep' any sane patient against his will in any home for six seconds, let alone for six weeks."

The words "against his will" are a qualification of the accusation in my original letter. I did not use them. The question of "will" does not arise; it is a question of judgment. A patient can be kept in a home or anywhere else by the doctor for just as long as his faith in the latter continues. If a patient has that which is an essential element in healing—faith in the healer—his independent power of judgment lies dormant. It is only when his faith begins to fail that his judgment begins to operate. And the process is slow. It takes quite a considerable time for the vague instinct of the patient that all is not well with him to crystallise into certain knowledge that the highly trained and self-confident physician from the purlieus of Harley Street is doing him more harm than good. There is a sort of religious atmosphere in the temple where the high priest of medicine performs his rites which does not exist in the freer air of an unconsecrated building, and which is a potent influence in retarding the growth of the spirit of unbelief.

Until the revelation comes the patient is kept secure in the nursing home bound by the fetters of his faith in the doctor.

I do not think it can be argued that doctors are unaware of the existence of this faith. They know it to be the most valuable curative agent they possess. The "system" I am trying to expose is based upon it.

But it is not perhaps until the account for professional services is rendered that the patient begins to realise that he has been in the grip of an organised "system." His eyes are then opened and much that was unintelligible becomes clear to him. He calculates the money value of, say, five patients for fifty weeks at six guineas a week each. He sees that if a doctor can gather a number of his patients into one building he does not waste time—which is money—in driving about the streets of London. He sees how yet more time—and more money—is saved to the doctor by the avoidance of lengthy explanations to nurses and cooks in private houses if he can induce his patients to enter an establishment where he presses a button and the staff does the rest. It dawns upon him that the doctor's preference for a particular nursing home is prompted by an ulterior motive which can be best expressed in terms of £ s. d.

The concluding paragraphs of your article while expressing sympathy with the resentment I feel against my doctor take me to task for seeking to discredit nursing homes. Certainly I had no such intention, and I do not see how my letter can have conveyed this impression. My sole object was to show how it is possible for a doctor to utilise a nursing home as an innocent accomplice in an ingenious variation of the confidence trick.—Yours faithfully,

